# Exploring the genetic architecture of specialized metabolism in Arabidopsis seeds

**DOI:** 10.1093/plphys/kiad554

**Published:** 2023-10-25

**Authors:** Henryk Straube

**Affiliations:** Assistant Features Editor, Plant Physiology, American Society of Plant Biologists; Faculty of Science, Department of Plant and Environmental Sciences, Section for Plant Biochemistry, University of Copenhagen, 1871 Frederiksberg C, Copenhagen, Denmark

Seeds play a fascinating role in the life cycle of seed plants (*Spermatophyta*). They contribute to the distribution of the offspring of land plants (Linkies et al. 2010) and provide as much as 70% of the worldwide calories for human nutrition (Sreenivasulu and Wobus 2013).

In addition to storing nutrients, seeds accumulate specialized metabolites with protective properties against various biotic and abiotic stresses (Debeaujon et al. 2000). Specialized metabolites can be valuable phytochemicals for human nutrition, but many have anti-nutritional properties (Scharff et al. 2022). To specifically modify the specialized metabolite content in seeds to increase nutritional value, comprehensive knowledge about their biosynthesis pathways is needed.

Coexpression analysis or gene-to-metabolite correlation analysis is often used to uncover genes involved in metabolite biosynthesis (Jacobowitz and Weng 2020). A powerful, yet arguably underexplored alternative for discovery of specialized metabolite pathway genes is genome-wide association studies (GWAS), which correlate genetic variants in an experimental population with certain traits, like the abundance of a metabolite (Fang and Jie 2019). In recent studies, researchers have used metabolic GWAS (mGWAS) to characterize the natural variation of specialized metabolism in rice (*Oryza sativa*; Chen et al. 2014; Matsuda et al. 2015) and maize (*Zea mays*; Zhou et al. 2019).

In this issue of *Plant Physiology*, Naake et al. (2023) performed an mGWAS for polar and semi-polar seed and leaf metabolites on a panel of 315 Arabidopsis (*Arabidopsis thaliana*) natural accessions (Fig. 1). Additionally, the authors performed quantitative trait locus (QTL) mapping for specific specialized metabolites in seeds.

**Figure 1.**
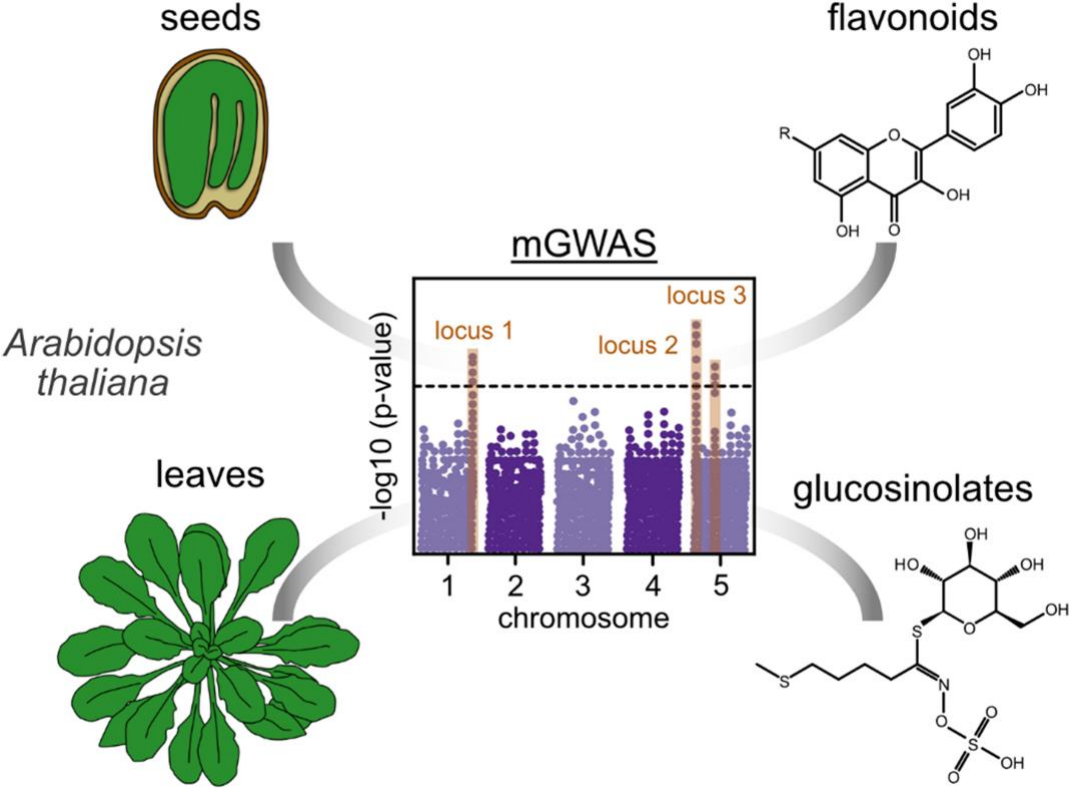
A schematic overview of the conducted experiment. Naake et al. (2023) performed a metabolic genome-wide association study (mGWAS) with metabolic features from leaves and seeds of Arabidopsis. They identified loci in the genome of Arabidopsis that are associated with flavonoids (represented by a quercetin molecule with a variable R) and glucosinolates (represented by 4-methylthiobutyl glucosinolate).

To identify metabolic features in leaves and seeds of the natural Arabidopsis accessions, the authors executed an untargeted metabolic profiling analysis using liquid chromatography coupled with high-resolution mass spectrometry. Using these data to perform mGWAS, they identified several conserved loci that control the abundance of metabolic features across different tissues.

The researchers annotated a subset of metabolic features based on previous studies, including glucosinolates and flavonoids, 2 well-studied classes of specialized metabolites.

For glucosinolates, 3 known loci were found to explain most of the variation in levels among different accessions, consistent with earlier QTL studies. Excitingly, the abundances of 2 unknown sulfur-containing compounds tentatively annotated as glucosinolates were also linked to these loci.

For flavonoids, the scientists identified 3 mass features that were putatively annotated as glycosylated quercetin-containing flavonols. GWAS revealed a linkage of these features with 2 genes encoding putative UDP-glucosyltransferases. To validate these findings, the authors characterized loss-of-function mutants in both genes. Only the mutant of the gene *AT5G17050*, encoding UDP-GLUCOSYL TRANSFERASE 78D2, showed differences in quercetin-containing flavonols in seeds.

Through GWAS and QTL mapping, Naake and colleagues (2023) harnessed the genetic diversity present in natural Arabidopsis populations and parental lines to investigate specialized seed metabolism. The resulting GWAS dataset is a high-quality community resource waiting to be explored in future research endeavors. As previously shown, the work of Naake et al. (2023) demonstrates the powerful complementarity of GWAS and QTL mapping.

GWAS and QTL mapping are promising alternatives when classic techniques like coexpression and gene-to-metabolite analyses fail to uncover genes involved in specialized metabolite biosynthesis pathways. With costs for genome sequencing decreasing constantly, it will soon be feasible to apply these approaches to many non-model plants. Additionally, mGWAS studies will contribute significantly to our understanding of metabolic diversity and its genetic basis.

## Dive Curated Terms

The following phenotypic, genotypic, and functional terms are of significance to the work described in this paper:

At5g17050 Gramene: AT5G17050
At5g17050 Araport: AT5G17050
